# Caregiver influence on looking behavior and brain responses in prelinguistic development

**DOI:** 10.3389/fpsyg.2014.00297

**Published:** 2014-04-11

**Authors:** Heather L. Ramsdell-Hudock

**Affiliations:** Infant Vocal Development Laboratory, Communication Sciences and Disorders, Idaho State UniversityPocatello, ID, USA

**Keywords:** looking behavior, brain response, prelinguistic vocal development, caregiver-infant interaction, audiovisual speech integration

Much is known about the development of speech perception, but there remain gaps in understanding in the development of speech production. Social interaction is a popular topic among researchers of human development, and the development of speech production is clearly dependent upon social interaction (Goldstein et al., 2003; Goldstein and Schwade, 2008). The importance of social interaction becomes particularly apparent when infants begin to speak the language of their ambient environment (Lewkowicz and Hansen-Tift, 2012). Prior to first word production, however, an exchange between caregivers and infants occurs, such that infant vocalizations and other changes in development directly influence caregiver responses, which in turn influence various components of interaction.

## Looking behavior and brain response

When presented with audiovisual stimuli of speaking faces between 6 and 12 months of age, researchers have compiled information on which parts of the face infants scan (e.g., around the eyes or the mouth), areas of brain activation, and associations between looking behavior and brain responses with later expressive and receptive language abilities. For looking behavior, a developmental shift in attention to audiovisual speech has been demonstrated between 4 and 8 months of age, with an increase in the time spent looking at the mouth as compared to eyes (Lewkowicz and Hansen-Tift, 2012). Increased looking behavior toward the mouth may support understanding of language, as faces convey an abundance of linguistic information (Wagner et al., 2013). Longer time looking to the eyes may indicate searching for additional social cues to resolve ambiguity in audiovisual stimuli (Kushnerenko et al., 2013).

For brain responses, significant negative correlations have been observed between auditory comprehension scores and the amplitude of the infantile P2 over right frontocentral brain regions in response to ambiguous audiovisual speech stimuli. The correlation weakens during the first year of development, presumably as auditory recognition improves. Infants with less precise, or less developed auditory speech processing at 6–9 months of age may rely more heavily on visual cues when ambiguous speech stimuli are presented (Kushnerenko et al., 2013). An alternative hypothesis is that infants may begin to attend more to the mouth region of a communication partner and process audiovisual information outside of the right lateralized frontocentral brain regions (Kushnerenko et al., 2013).

## Prelinguistic vocal development and caregiver-infant interaction

The shift in visual attention may also be related to vocal development. In particular, developmental advances occurring with respect to speech production between 6 and 12 months of age directly influence caregiver interaction. Caregivers begin to attend more, and respond differently to well-formed infant productions. This in turn, may lead infants to allocate visual attention to different regions of the face, and hence, encourage the eyes-to-mouth-to-eyes attentional shift.

Infants normally transition through several stages of vocal development in the first year of life. In the phonation stage, from birth to 2 months of age, infants gradually become more able to manipulate normal phonation in production of quasivowels. In the primitive articulation stage, from 1 to 4 months of age, infants gradually become more capable of manipulating their vocal tract during voicing in production of “cooing” and “gooing” sounds. In the expansion stage, from 3 to 8 months of age, infants gradually become more able to open and posture their vocal tract in production of full vowels and marginal babbles (Oller, 2000). During these first months of life, caregivers are likely to simply gauge infant well-being from phonation, and to imitate vocalizations produced by the infant (Julien and Munson, 2012; Olson and Masur, 2012). This sort of action from the caregiver does not enhance vocalizations and may not draw the infant's attention toward the mouth—the visible part of the speech mechanism.

In the canonical stage, from approximately 5–10 months of age, infants gradually become more able to make well-timed movements of their articulators from open to closed postures in production of canonical babbling (Oller, 2000). In this final stage, prior to production of words, infants begin to produce syllables, potential components of words (Oller, 1980; Koopmans-van Beinum and van der Stelt, 1986; Stark et al., 1993). When caregivers identify these babbled syllables, they intuitively begin to interact with the infant around them, treating them as potential words (Veneziano, 1988; Stoel-Gammon, 2011). As word learning begins, caregivers engage infants, recognizing babbled syllables and their potential relation with the ambient language (Ramsdell et al., 2012; Oller et al., 2013). In this stage, infant vocalizations are well-formed and more familiar, and caregivers begin to encourage language growth through expanding on and enhancing these productions (Gros-Louis et al., 2006; Olson and Masur, 2012). Simultaneously, infants are learning from caregiver input. In a study conducted by Goldstein and Schwade, sixty 9.5 month old infants were found to begin producing more speech-like forms in coordination with caregiver response to their vocalizations (2008). At this point in development, caregiver response to infant vocalizations may draw the infant's attention toward the mouth, and the infant then receives multimodal (auditory and visual) feedback supporting productions, which in turn help to facilitate vocal development.

Still, there are other interpretations that may also contribute to shifts in attention. Perhaps not only changing caregiver/infant interaction, but also changing social-cognitive abilities for joint attention later in the first year of development, shift attention from the mouth, back to the eyes. Infants engage in more joint attention episodes between 9 and 12 months of age, with improving skill for triadic interactions from people to objects (Bakeman and Adamson, 1984). Further, infants are utilizing new forms of nonverbal communication during interactions, such as pointing, showing, and giving (Bates et al., 1975). During this time, joint attention and gesture use occur together with coordinating eye gaze between objects and social partners (Messinger and Fogel, 1998; Wu and Gros-Louis, 2014).

## Conclusion

As prelinguistic vocalizations develop and become well-formed, caregiver responses change to *teach* language. This changing caregiver-infant interaction could help to guide the infant in attending to different areas of the face, thereby causing processing to shift to different neural circuitry. Accordingly, the changes occurring in looking behavior and brain responses are likely not to be solely dependent upon the infants own endogenous attentional mechanisms and motivations to vocalize, but dependent on caregiver interaction as well.

### Conflict of interest statement

The authors declare that the research was conducted in the absence of any commercial or financial relationships that could be construed as a potential conflict of interest.
